# Association of Serum Adipokines and Resting Energy Expenditure in Patients With Chronic Kidney Disease

**DOI:** 10.3389/fnut.2022.828341

**Published:** 2022-03-15

**Authors:** Nanzha Abi, Xiao Xu, Zhikai Yang, Tiantian Ma, Jie Dong

**Affiliations:** ^1^Renal Division, Department of Medicine, Peking University First Hospital, Beijing, China; ^2^Institute of Nephrology, Peking University, Beijing, China; ^3^Key Laboratory of Renal Disease, Ministry of Health, Beijing, China; ^4^Key Laboratory of Renal Disease, Ministry of Education, Beijing, China; ^5^Research Units of Diagnosis and Treatment of Immune-Mediated Kidney Diseases, Chinese Academy of Medical Sciences, Beijing, China

**Keywords:** chronic kidney disease, resting energy expenditure, leptin, adiponectin, interleukin 6, adipokines, body composition

## Abstract

**Background and Aim:**

Metabolic disorders are prevalent in patients with chronic kidney disease (CKD) and may lead to protein energy wasting (PEW). Adipokines improve connections between PEW and energy metabolism. We aimed to determine the relationship between adipokine levels and resting energy expenditure (REE) in patients with CKD.

**Methods:**

A total of 208 patients in non-dialyzed CKD stages 3–5 were enrolled in this cross-sectional study. Serum adipokines (leptin, adiponectin, and interleukin 6 (IL-6) were measured using enzyme-linked immunosorbent assay. Patient's REE was measured using indirect calorimetry. Fat mass (FM) and lean tissue mass (LTM) were measured using multiple-frequency bioimpedance analysis. Spearman correlation analyses and multivariate linear regression models were used to assess the association between serum adipokines and REE.

**Results:**

The mean age was 52.7 ± 14.6 years, and 26.9, 26.4, and 46.7% of our participants had CKD stages 3, 4, and 5, respectively. The median values of serum adiponectin, leptin, and IL-6 were 470.4 (range, 291.1–802.2), 238.1 (range, 187.9–418.4), and 4.0 (range, 2.4–9.5) pg/mL, respectively. The male participants had significantly lower FM% (*P* = 0.001) and lower leptin levels (*P* < 0.001) than the female participants. After adjusting for age, diabetes, high-sensitivity C-reactive protein, intact parathyroid hormone, LTM, and FM, multiple linear regression analysis revealed that serum leptin levels were significantly positively associated with REE in men rather than in women (*P* < 0.05). Serum adiponectin levels were inversely associated with REE in men, but this association disappeared while FM was additionally adjusted. Adiponectin levels in women were not correlated with REE (*P* > 0.05). IL-6 was not significantly associated with REE in either men or women.

**Conclusions:**

A sex-specific relationship between serum adipokines (leptin and adiponectin) and REE was observed in patients with CKD stages 3–5, which was partly confounded by FM.

## Introduction

Individuals with chronic kidney disease (CKD) are predisposed to protein energy wasting (PEW) owing to various pathophysiological factors (1, 2), with the prevalence of 18 to 48% in patients with CKD stages 3–4 and reach as high as 75% in patients with CKD stage 5 (3), finally resulting in poor clinical outcomes and reduced quality of life (4). As a major component of wasting syndrome, altered energy expenditure is prominent and closely related to renal function, because kidney accounts for about 10% of REE (5, 6). Although key factors such as age, comorbidities, and body composition were evaluated, we cannot fully explain the individualized energy expenditure for a specific patient (7). More potential mechanisms for energy hemostasis deserve further investigation in the CKD population.

Adipokines, namely adipocyte-enriched regulatory peptides, are mainly secreted by adipose tissue (8). Adipokines are known to play an important role in energy metabolism. Elevated levels of leptin (9), adiponectin (10), and interleukin-6 (IL-6) (11), are markers of kidney injury and risk of disease progression as well as modulating factors of energy expenditure, appetite, glucose metabolism, and lipid metabolism (8, 12, 13). However, current evidence on the association of these adipokines with energy expenditure in the general population and individuals with chronic disease has shown inconsistent findings (14–18). Adipokines interventions have been studied to ameliorate weight loss-induced changes (19, 20), cachexia from CKD (21), and cancer (22, 23), but evidence, which is based on animal models or small-sample clinical trials, is still preliminary. Before adipokines are administered as a promising intervention to modulate wasting syndrome in CKD patients, we should fully explore its associations with resting energy expenditure (REE).

Therefore, we aimed to explore the independent relationship between circulating adipokines (leptin, adiponectin, and IL-6) and REE. Of note, body components including fat mass (FM) and lean tissue mass (LTM) are key contributors of REE (24). The distribution of body composition differs between sexes in CKD (25). More importantly, FM *per se* is closely associated with adipokines levels (26, 27). Thus, we constructed models for analyzing the association of adipokines and REE in male and female participants, respectively, using both FM and LTM as key confounders. Our results would be helpful to uncover the connection between adipokines and REE in CKD.

## Materials and Methods

### Study Design and Patients

This is a *post hoc* analysis of the study on a novel equation for estimating REE in CKD patients (7). The study recruited outpatients with CKD according to the following inclusion criteria: age ≥18 years; non-dialyzed with CKD stages 3–5; consented to participate in all aspects of the study; willing to provide serum samples. Patients with the following conditions were excluded: abnormal thyroid function; a history of amputation; pregnancy; corticosteroid or immunosuppressive medication; comorbidities associated with protein catabolism, such as acute or chronic systemic infections, acute cardiovascular events, operations, trauma, an acute episode of gout within the previous 4 weeks, or tumors for which a patient had received radiotherapy or chemotherapy within 6 months; lung diseases that affected the measurement of gas exchange and body metabolism, such as asthma, chronic obstructive pulmonary disease, pneumothorax, and pleural effusion. The Ethics Committee of Peking University First Hospital approved the study protocol and adhered to the Declaration of Helsinki. Each patient provided written informed consent to participate in the study. This trial was registered at ClinicalTrials.gov (NCT03377413).

### Demographic and Laboratory Measurements

Demographic and clinical data including age, sex, height, weight, primary renal disease, and diabetes mellitus (DM) were collected. Standing height was measured using a fixed stadiometer, and weight was measured using a calibrated digital scale.

Blood samples were collected following an overnight fast. Biochemistry data in relation to hemoglobin, serum albumin, lipids, glucose, uric acid, urea, creatinine, calcium, and phosphate were obtained using an automatic chemistry analyzer (Hitachi Chemicals). The estimated glomerular filtration rate (eGFR) was calculated using the Chinese equation for CKD patients (28). Serum concentrations of high-sensitivity C-reactive protein (hs-CRP) were measured using immune rate nephelometry (normal values, <3 mg/L). Serum intact parathyroid hormone (iPTH) levels were measured using a chemiluminescence assay (reference range, 15–65 pg/mL).

### Adipokines Measurements

Serum concentrations of leptin and adiponectin were measured using enzyme-linked immunosorbent assay (ELISA) method (eBioscience, San Diego, CA, USA), with sensitivities of 6.4 and 56 pg/mL, respectively. IL-6 level was measured using commercially available ELISA kits (Beckmann Coulter Inc. Brea, CA, USA), with a sensitivity of 0.5 pg/mL. The normal IL-6 value is <6.5 pg/mL.

### Body Composition

Multiple-frequency bioimpedance analysis was performed (BCM; Fresenius Medical Care, Bad Homburg, Germany); this procedure has been described in detail elsewhere (29), Briefly, with the patient positioned supine for a minimum of 10 min, standard tetrapolar electrodes were placed on the dorsal surface of the left wrist and on the anterior aspect of the left ankle. Three consecutive measurements were performed during a 2-min period, and the values of extracellular water (ECW), intracellular water (ICW), and total body water (TBW) were recorded. Based on these data, FM and LTM were estimated.

### Resting Energy Expenditure

REE was measured using indirect calorimetry (IC) with a VMax 29 n metabolic cart (CareFusion, Yorba Linda, CA, USA). The patients fasted overnight (>12 h). After 30 min of rest, they completed the measurements between 08:00 and 11:00 AM in a quiet, dimly lit room maintained at a constant humidity (room temperature, 20–25°C). During the test, the patients were instructed to lie supine for 15 min, breathe calmly, and avoid hyperventilation, fidgeting, or falling asleep. Oxygen consumption and carbon dioxide production were measured at 30-s intervals. Data were recorded only when the patients were in steady-state conditions, and the average O_2_ and CO_2_ volumes were used to calculate REE using the Weir equation (30).

### Statistical Analysis

Normally distributed data are presented as mean ± standard deviation. Non-normal data are presented as median values with interquartile range. Categorical variables were expressed as percentages or ratios. Student's *t*, non-parametric, or χ^2^ tests were used to compare the differences between male and female participants. Spearman correlation analyses were used to ascertain the relationship between various variables (all demographic and biochemical measurements) and REE; subsequently, the significant factors (age, DM, hs-CRP, and iPTH) were applied to a multivariate linear regression model to investigate the associations between serum leptin, adiponectin, and IL-6 and REE. Considering that the LTM and FM are components of body weight, we adopted three models to explore the independent effects of adipokines on REE: model 1 was adjusted for age, DM, hs-CRP, and iPTH; model 2 was adjusted for age, DM, hs-CRP, iPTH, and LTM; model 3 was adjusted for age, DM, hs-CRP, iPTH, and FM. Because IL-6 and hs-CRP are both inflammatory cytokines, hs-CRP was not adjusted in the three models with IL-6 as the independent variable. Because the distributions of serum adiponectin, leptin, and IL-6 levels were skewed, we used the log-transformed values of these variables in the regression analyses.

All probabilities were two-tailed, and the level of significance was set at 0.05. Statistical analysis was performed using SPSS for Windows software version 21.0 (IBM Corp., Armonk, NY, USA).

## Results

### Basic Characteristics

**Figure 1:**
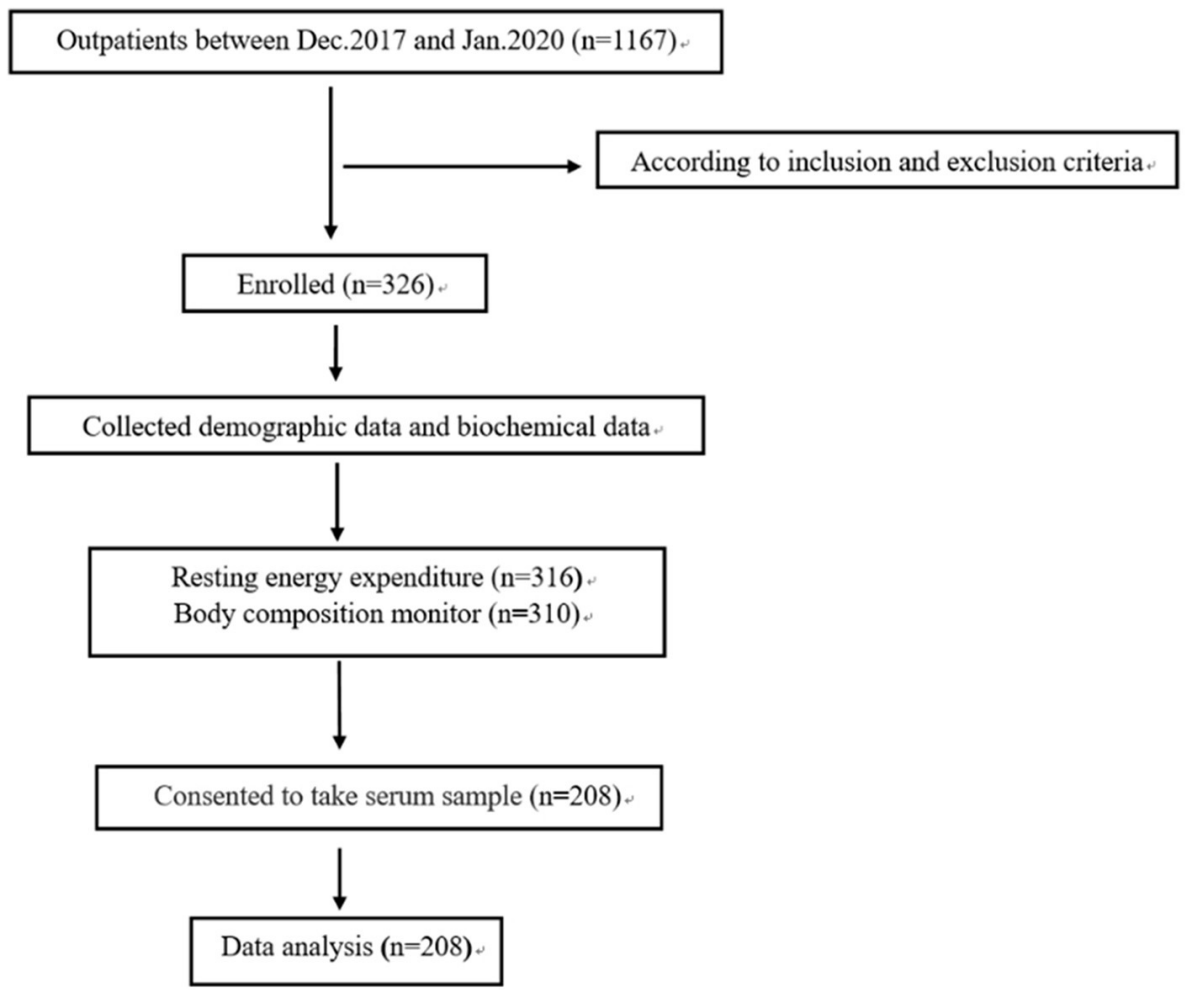
Flow chart of the study.

Of the 326 patients recruited, 208 patients with stage 3–5 non-dialyzed CKD were included in the final study (Figure 1). The baseline characteristics of the patients are shown in Table 1. The basic characteristics showed that the mean age was 52.7 ± 14.6 years, 130 patients were men, and 72 (33%) had DM, which are proportional to the characteristics of the general CKD population in China published previously (31). The distribution of CKD stages was 26.9, 26.4, and 46.7% for stages 3, 4, and 5, respectively. The mean BMIs were 24.8 ± 4.2 kg/m^2^, and 44.7% of the patients were overweight. The mean serum albumin and hemoglobin levels were normal, with low hs-CRP values, representing a relatively stable status.

**Table 1:** Demographic and clinical characteristics of CKD patients in male and female groups.

**Variates**	**All (***n*** = 208)**	**Male (***n*** = 130)**	**Female (***n*** = 78)**	***P*** **-value**
Age, y	52.7 ± 14.6	52.0 ± 15.2	53.8 ± 13.5	0.4
DM, *n* (%)	72 (33.0)	54 (41.5)	18 (23.0)	0.007
CKD stage, *n* (%)				0.881
3	56 (26.9)	33 (25.4)	23 (29.5)	
4	55 (26.4)	35 (26.9)	20 (25.6)	
5	97 (46.7)	62 (47.7)	35 (44.9)	
Height, cm	164.7 ± 8.46	169.2 ± 6.55	157.2 ± 5.36	<0.001
Weight, kg	67.7 ± 15.1	73.0 ± 15.0	58.8 ± 10.4	<0.001
BMI, kg/m^2^	24.8 ± 4.20	25.4 ± 4.33	23.8 ± 3.79	0.007
**Laboratory data**				
Serum albumin, g/L	40.9 ± 4.44	40.8 ± 4.76	41.0 ± 3.87	0.775
Hemoglobin, g/L	116 ± 20.2	119.3 ± 21.1	110.4 ± 17.4	0.001
hs-CRP, mg/L	1.20 (0.50, 2.50)	1.17 (0.51, 2.5)	1.22 (0.6, 2.41)	0.767
Urea nitrogen, mmol/L	17.5 (22.9, 23.8)	19.9 ± 9.4	15.4 (10.3, 22.7)	0.022
Serum creatinine, μmol/L	319.5 (191.5, 542.6)	347 (230.8, 554.8)	260.5 (154.3, 523.3)	0.032
Serum calcium, mmol/L	2.29 ± 0.16	2.27 ± 0.17	2.31 ± 0.15	0.162
Serum phosphorus, mmol/L	1.44 ± 0.40	1.43 ± 0.43	1.46 ± 0.36	0.578
Serum sodium, mmol/L	140.3 ± 2.71	140.4 ± 2.41	140.1 ± 3.14	0.398
Serum potassium, mmol/L	4.57 ± 0.58	4.55 ± 0.59	4.58 ± 0.57	0.735
Total cholesterol, mmol/L	4.61 ± 1.19	4.39 ± 1.06	4.97 ± 1.31	0.01
Triglycerides, mmol/L	1.59 (1.16, 2.30)	1.64 (1.15, 2.36)	1.53 (1.19, 2.20)	0.738
iPTH, pg/mL	127.8 (72.5, 253.2)	133.5 (79.2, 256.0)	113.1 (65.2, 249.0)	0.957
eGFR, mL/min/1.73 m^2^	16.4 (8.9, 30.5)	16.5 (9.28, 30.2)	16.4 (7.58, 32.4)	0.775

*DM, diabetes mellitus; CKD, chronic kidney disease; BMI, body mass index; hs-CRP, high-sensitivity C-reactive protein; iPTH, intact parathyroid hormone; eGFR, estimated glomerular filtration rate*.

*Data are expressed as mean ± SD or median values with their lower and upper quartiles for numerical variables and percentage or ratio for categorical variables*.

The height, weight, BMI, and percentage of DM were significantly higher in men than in women (*P* < 0.01 for all). Despite the comparable eGFR and distribution of CKD stages between sexes, the male participants had significantly higher hemoglobin, urea nitrogen, and serum creatinine but lower cholesterol values (*P* = 0.001, *P* = 0.022, *P* = 0.032 and *P* = 0.01, respectively).

### Serum Adipokines, Body Composition, and REE

The distributions of serum adiponectin, leptin, and IL-6 were skewed. As shown in Table 2, the median values of serum adiponectin, leptin, and IL-6 were 470.4 (291.1, 802.2), 238.1 (187.9–418.4), and 4.0 (2.4–9.5) pg/mL, respectively, which were similar to those in other CKD samples (32). Men had slightly lower adiponectin and significantly lower leptin levels (*P* = 0.054 and *P* < 0.001, respectively), but their serum IL-6 levels were comparable with those of women (*P* = 0.383).

**Table 2:** Serum adipokines, body composition and REE in male and female groups.

**Variates**	**All (***n*** = 208)**	**Male (***n*** = 130)**	**Female (***n*** = 78)**	***P*** **-value**
ECW, L	16.3 ± 3.53	18.0 ± 3.13	13.5 ± 2.13	<0.001
ICW, L	19.2 ± 5.16	21.6 ± 4.97	15.3 ± 2.32	<0.001
TBW, L	35.5 ± 8.16	39.6 ± 7.29	28.8 ± 4.11	<0.001
LTM, kg	39.5 ± 10.3	44.5 ± 9.06	31.1 ± 5.85	<0.001
LTM%	59.8 ± 13.0	63.3 ± 12.5	54.1 ± 11.8	<0.001
FM, kg	19.6 ± 9.2	19.6 ± 10.6	19.6 ± 7.70	0.957
FM%	28.5 ± 13.7	26.3 ± 15.6	32.2 ± 8.70	0.001
Adiponectin, pg/ml	470.4 (291.1, 802.2)	416.6 (266.9, 722.3)	553.8 (346.1, 854.9)	0.054
Leptin, pg/ml	238.1 (187.9, 418.4)	216.4 (180.0, 287.2)	350.7 (215.7, 609.7)	<0.001
IL-6, pg/ml	3.98 (2.42, 9.52)	4.09 (2.44, 10.1)	3.76 (2.32, 7.31)	0.383
REE, kcal/d	1374.2 ± 296.8	1498.2 ± 290.2	1167.5 ± 163.0	<0.001

*ECW, extracellular water; ICW, intracellular water; TBW, total body water; LTM, lean tissue mass; FM, fat mass; IL, Interleukin; REE, resting energy expenditure*.

With regards to body composition, males had significantly higher ECW, ICW, TBW and LTM (*P* < 0.001 for all) but comparable FM compared with females (*P* = 0.957), a lower FM% calculated accordingly (*P* = 0.001). Due to a larger body size, REE values were also significantly higher in male patients (*P* < 0.001). Above findings are shown in Table 2.

**Figure 2:**
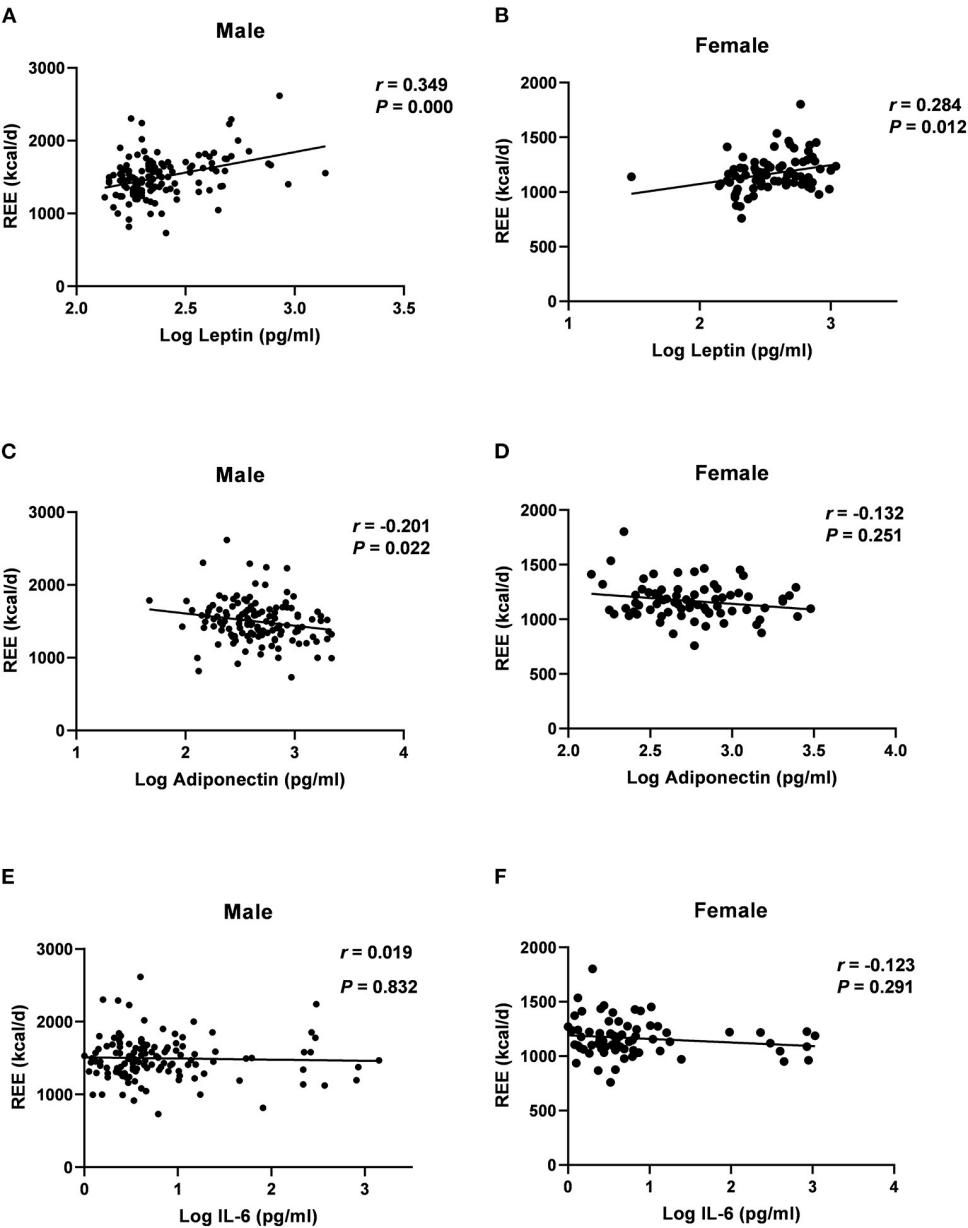
Correlations of log-transformed adipokines level with REE in males and female groups, assessed by Log leptin **(A,B)**; Log adiponectin **(C,D)**; Log IL-6 **(E,F)**; REE, resting energy expenditure; IL, Interleukin; r, Spearman's rank correlation coefficient.

Spearman correlation analyses showed that serum levels of log-transformed leptin were positively associated with REE in men (*r* = 0.349, *P* = 0.000) and women (*r* = 0.284, *P* = 0.012) (Figures 2A,B). Log-transformed serum adiponectin levels were negatively correlated with REE in men (r = −0.201, *P* = 0.022) (Figure 2C), but no significant difference was found in women (*P* = 0.251) (Figure 2D). Log-transformed IL-6 was not significantly associated with REE in either men or women (Figures 2E,F) (*P* > 0.05). Table 3 listed the correlation coefficients between other variables and REE.

**Table 3:** Correlation Coefficients between variables and REE.

**Variable**	**Total**
Age	−0.24**
DM	0.20**
LTM	0.72**
FM	0.34*
Hemoglobin	0.09
Serum albumin	−0.05
Urea nitrogen	0.15*
Serum creatinine	0.21**
Serum potassium	0.03
Serum calcium	−0.07
Serum sodium	−0.08
Serum phosphate	0.10
Triglycerides	0.28**
Total cholesterol	−0.10
hs-CRP	0.19**
iPTH	0.14*

*REE, resting energy expenditure; DM, diabetes mellitus; LTM, lean tissue mass; FM, fat mass; hs-CRP, high-sensitivity C-reactive protein; iPTH, intact parathyroid hormone*.

* *P < 0.05 and*

** *P < 0.01 for correlation coefficients between variables*.

We performed multivariate linear regression analyses to examine the relationships between log-transformed serum adiponectin, leptin, and IL-6 and REE (Table 4). For men, adiponectin was negatively associated with REE in models 1 and 2 (*P* = 0.02 and *P* = 0.013, respectively). Nevertheless, when FM was introduced into the regression model, the effect of adiponectin levels on REE disappeared. There was a positive association between leptin level and REE after adjusting for age, DM, hs-CRP, and iPTH (*P* < 0.001). This association did not change even after further adjusting for LTM or FM (*P* < 0.001 and *P* <0.031, respectively). For women, only leptin, rather than adiponectin, showed a significantly positive association with REE in models 1 and 2 (*P* = 0.024 and *P* = 0.002, respectively). When FM was introduced into the model, leptin did not retain its significant association with REE (*P* = 0.186). For both men and women, IL-6 levels were not associated with REE in any of the models (*P* > 0.05).

**Table 4:** Multiple linear regressions of serum adipokines and REE for males and females.

**Variable**	**Male**			**Female**		
	**β**	**95%CI**	***P***	**β**	**95%CI**	***P***
**Leptin**						
Model 1	0.33	(0.17, 0.48)	<0.001	0.25	(0.04, 0.47)	0.024
Model 2	0.28	(0.15, 0.43)	<0.001	0.28	(0.10, 0.45)	0.002
Model 3	0.17	(0.02, 0.33)	0.031	0.15	(−0.08, 0.38)	0.186
**Adiponectin**						
Model 1	−0.19	(−0.36, −0.03)	0.020	−0.12	(−0.36, 0.12)	0.329
Model 2	−0.18	(−0.33, −0.04)	0.013	−0.13	(−0.33, 0.07)	0.211
Model 3	−0.08	(−0.23, 0.07)	0.275	0.01	(−0.24, 0.24)	0.995
**IL-6**						
Model 1	0.01	(−0.16, 0.17)	0.930	−0.18	(−0.41, 0.06)	0.136
Model 2	0.01	(−0.14, 0.16)	0.904	−0.14	(−0.34, 0.05)	0.152
Model 3	−0.04	(−0.18, 0.10)	0.592	−0.21	(−0.43, 0.02)	0.067

*Model 1 was adjusted for age, DM, hs-CRP, iPTH*.

*Model 2 was adjusted for age, DM, hs-CRP, iPTH, LTM*.

*Model 3 was adjusted for age, DM, hs-CRP, iPTH, FM*.

*hs-CRP was not adjusted in 3 models with IL-6 as the independent variable; Adiponectin, leptin, IL-6 were log-transformed for analyses*.

## Discussion

This study explored the association between serum adipokines, such as leptin, adiponectin, and IL-6 levels, and REE values measured by IC, the gold standard of REE, in patients with CKD stages 3–5 through a single-center cross-sectional study. Our data indicated that serum leptin and adiponectin were closely correlated with REE in all or only in male participants. The association of serum leptin, adiponectin and REE was in part confounded by the FM. Serum IL-6 levels were not associated with the REE in either unadjusted or adjusted models.

As shown in our data, serum leptin exerted an independent effect on REE after multivariate adjustment in our male participant. The correlation coefficients of serum leptin and REE are similar to those reported in the general population (14). As a peripheral signal that informs the brain of the metabolic state, it is believed that leptin increases REE through its effects on the cardiovascular system and brown adipose tissue thermogenesis *via* the hypothalamus (33). A positive association between REE and leptin was observed in cross-sectional studies involving 40 to 50 patients with chronic obstructive pulmonary disease (COPD) (17) or heart failure (HF) (27). Leptin administration in obese or normal participants increased energy expenditure and food intake. Conversely, leptin receptor antagonists reduced REE in patients with lipodystrophy (34). CKD-associated cachexia and PEW in rats were ameliorated by a leptin antagonist (21, 35), or by blockade of the leptin receptor (36). Our data support an independent relationship between serum leptin and REE in CKD, especially in men. In this context, we hope the relationship of leptin with energy expenditure in CKD patients will provide clues for blocking leptin activity as a novel therapeutic strategy for PEW in CKD. Of note, about half patients were overweighted in our study, supporting special phenomenon of obese sarcopenia in CKD population. We should observe if leptin antagonists would exacerbate obesity while energy catabolism is corrected in this population.

In contrast, our data did not show a significant independent association between serum adiponectin and REE in our CKD patients. Adiponectin is considered to play a health-promoting role in metabolic homeostasis, such as increasing fatty acid oxidation in the liver and skeletal muscle or reducing liver gluconeogenesis and inflammation (37), which suggests a potential modulator role in energy expenditure. Adiponectin injection in mice has been reported to stimulate food intake and reduce energy expenditure (38). However, observational studies involving patients with COPD, HF, and obesity have shown inconsistent findings on the association between REE and adiponectin values (17, 27, 39). On the basis of myriad pathologies of diseases, we need to explore whether using adiponectin as an agonist or antagonist can modulate energy expenditure in each specific disease. Although our data did not support an independent effect of adiponectin on energy expenditure, we still cannot exclude the possibility that adiponectin plays a role in energy balance through its modulating effect via other pathways (e.g., food intake) in CKD.

Our analysis suggested that a sex-specific relationship between serum adipokines and REE exists in CKD patients. The association between energy expenditure and serum adipokines (leptin and adiponectin) was much weaker in female patients. To the best of our knowledge, there is a different complex distribution of adipokines across sex, which could lead to distinct downstream biological effects (40–42). In addition, sex hormones may influence the biological roles of adipokines. For example, one study indicated that estradiol (43) potentiates the anorexigenic action by enhancing leptin sensitivity within the brain, whereas androgen 5α-dihydrotestosterone seems to operate in the opposite manner (44). Another study suggested that testosterone may have direct effects on the modulation of production, complex formation, and clearance of adiponectin (45). Whether sex hormones can influence the systemic effects of adipokines via specific receptors in remote target organs has not been determined. Further research is required to explore how sexual dimorphism influences the effects of adipokines on energy expenditure.

As shown in our data, FM is a key confounder in the association between serum adipokines and REE. FM is closely linked to the concentration of adipose-derived hormones, including serum leptin and adiponectin (46, 47), which may in turn disturb the effect of adipokines on REE. In men, adiponectin was found to be negatively associated with REE, but this association disappeared after additional adjusting for FM. In women, leptin had a significantly positive association with REE, which was weakened after adjusting for FM again. Our findings are in line with previous reports that the inverse relationship between adiponectin and REE disappeared after adjusting for FM in adult women (48) and COPD patients (17). Similarly, a study showed a positive association between leptin and REE only in COPD patients before adjusting for FM (17). Given that leptin and adiponectin are the only two peptides that are selectively expressed in adipocytes (8), the impact of leptin on modulating the REE independent of FM, as shown in our data, can be proven as the endocrine hormone that exert systemic biological effects in CKD.

Inflammation is closely associated with increased REE in patients with CKD (49–51). The relatively high levels of inflammatory cytokines, such as IL-6, in patients undergoing hemodialysis (median, 7.1 pg/mL; range, 2.2–163.5 pg/mL) (52), those with lung cancer (mean, 30.3 ± 40.2 pg/mL) (15), and those with Crohn disease (mean, 13.8 ± 13.4 pg/mL) (53), show a positive correlation with REE. However, IL-6 appeared not to be associated with REE in our cohort, as has been reported in patients with HF (27). We consider that a relatively lower IL-6 level (median, 4.0 pg/mL; range, 2.4–9.5 pg/mL) in our cohort, compared with patients with lung cancer (15) and inflammatory-bowel disease (53), represents a stable clinical state, which may mask a potential relationship between IL-6 and REE.

This study had several strengths. For the first time, the relationship between adipokines, including leptin, adiponectin, and IL-6, and REE was analyzed in patients with CKD stages 3–5. Our findings provide preliminary evidence on the potential benefits of adipokines-related interventions on energy expenditure in this population. In addition, REE was measured using IC, the gold standard of REE. Another strength of our study was that the data of men and women were analyzed separately due to different fat mass distributions. The confounding effects of LTM and FM on the relationship between adipokines and REE were further evaluated in men and women, respectively. The sex difference in the regulation of adipokines in REE will encourage further exploration of sexual dimorphism in the association between adipokines and energy expenditure.

There are some limitations to our study. First, our cross-sectional study could not determine whether associations between adipokines and REE represent a causal process. The causal relationship between REE and adipokines needs to be examined in prospective and interventional studies. Second, body compositions (FM and LTM) were estimated using the BCM instead of dual-energy X-ray absorptiometry. We did not measure visceral or subcutaneous FM using CT or MRI, which is not helpful for differentiating the effect of regional FM on serum adipokines. The complex cross-talk among FM, serum adipokines, and REE still needs to be explored in further research.

## Conclusion

Our results suggest that serum leptin levels are positively associated with REE in male patients with CKD stages 3–5, providing evidence for the role of leptin in energy metabolism in this population. Moreover, a sex-specific relationship between serum adipokines (leptin and adiponectin) and REE was observed, which was, in part, confounded by FM. Further research is needed on how sexual dimorphism and the distribution of body composition influence the effects of adipokines on energy expenditure.

## Data Availability Statement

Data described in the manuscript, code book, and analytic code are not readily available because the Management of China's Human Genetic Resources does not allow sharing this information. Further inquiries can be directed to the corresponding author.

## Ethics Statement

The studies involving human participants were reviewed and approved by the Ethics Committee of Peking University First Hospital. The patients/participants provided their written informed consent to participate in this study.

## Author Contributions

JD and XX: research idea and study design. NA, XX, ZY, and TM: data acquisition. NA, XX, and JD: statistical analysis and manuscript drafting or revision. JD: supervision or mentorship. All authors: read and approved the final manuscript.

## Funding

This work is supported in part by the Scientific Research Project of Capital Health Development (2020-2-4079); New Century Excellent Talents from Education Department of China (BMU20110265); Clinic Research Award from ISN GO R&P Committee; CAMS Innovation Fund for Medical Sciences (2019-I2M-5-046).

## Conflict of Interest

The authors declare that the research was conducted in the absence of any commercial or financial relationships that could be construed as a potential conflict of interest.

## Publisher's Note

All claims expressed in this article are solely those of the authors and do not necessarily represent those of their affiliated organizations, or those of the publisher, the editors and the reviewers. Any product that may be evaluated in this article, or claim that may be made by its manufacturer, is not guaranteed or endorsed by the publisher.

## Abbreviations

BMI: body mass index
CKD: chronic kidney disease
COPD: chronic obstructive pulmonary disease
DM: diabetes mellitus
ECW: extracellular water
eGFR: estimated glomerular filtration rate
ELISA: enzyme-linked immunosorbent assay
FM: fat mass
HF: heart failure
hs-CRP: high-sensitivity C-reactive protein
IC: indirect calorimetry
ICW: intracellular water
IL-6: interleukin 6
iPTH: intact parathyroid hormone
LTM: lean tissue mass
PEW: protein energy wasting
REE: resting energy expenditure
TBW: total body water.
